# m6A regulator-mediated methylation modification patterns and tumor microenvironment immune infiltration with prognostic analysis in esophageal cancer

**DOI:** 10.1038/s41598-023-46729-1

**Published:** 2023-11-11

**Authors:** Gaohong Sheng, Tianqi Wang, Yuan Gao, Hua Wu, Jianhong Wu

**Affiliations:** 1 Department of Orthopedics, Tongji Hospital, Tongji Medical College, Huazhong University of Science and Technology, Jiefang Avenue 1095, Wuhan, China; 2 Department of Oncology, Tongji Hospital, Tongji Medical College, Huazhong University of Science and Technology, Jiefang Avenue 1095, Wuhan, China; 3 Third Hospital of Shanxi Medical University, Shanxi Bethune Hospital, Shanxi Academy of Medical Sciences, Tongji Shanxi Hospital, Taiyuan, China; 4 Gastrointestinal Surgery Center, Tongji Hospital, Tongji Medical College, Huazhong University of Science and Technology, Jiefang Avenue 1095, Wuhan, China

**Keywords:** Cancer, Computational biology and bioinformatics, Genetics, Molecular biology, Biomarkers, Oncology

## Abstract

Esophageal cancer is a highly malignant disease with poor prognosis. Despite recent advances in the study of esophageal cancer, there has been only limited improvement in the treatment and prognosis. N6-methyladenosine (m6A), a type of RNA modification, has been extensively investigated and is involved in many biological behaviors, including tumorigenesis and progression. Thus, more research on m6A modification may increase our understanding of esophageal cancer pathogenesis and provide potential targets. In our study, we integrated the public data of esophageal cancer from The Cancer Genome Atlas (TCGA) and Gene-Expression Omnibus (GEO) databases. Unsupervised clustering analysis was used to classify patients into different groups. Gene set variation analysis (GSVA) was performed in a nonparametric and unsupervised mode. We evaluated immune cell infiltration by single sample gene set enrichment analysis (ssGSEA). Differentially expressed genes (DEGs) among m6A clusters were identified using Empirical Bayesian approach. Both multivariate and univariate Cox regression models were used for prognostic analysis. We provided an overview of gene variation and expression of 23 m6A regulators in esophageal cancer, as well as their effects on survival. Based on the overall expression level of m6A regulators, patients were classified into three m6A clusters (A-C) with different immune cell infiltration abundance, gene expression signatures and prognosis. Among m6A clusters, we identified 206 DEGs, according to which patients were classified into 4 gene clusters (A-D). Quantitative m6A score was calculated for each patient based on those DEGs with significant impact on survival. The infiltration of all types of immune cells except type 2 T helper (Th2) cells were negatively correlated with m6A score. M6Acluster C exhibited the lowest m6A score, the most abundant immune cell infiltration, and the worst prognosis, suggesting an immune excluded phenotype. Consistently, gene cluster D with the lowest m6A score showed the worst prognosis. In short, patients with esophageal cancer showed different m6A modification patterns. Quantitative scoring indicated that patients with the lowest m6A score exhibited the most abundant immune cell infiltration and the poorest prognosis. This m6A scoring system is promising to assess m6A modification pattern, characterize immune infiltration and guide personalized treatment and prognostic prediction.

## Introduction

Esophageal cancer (EC) is a highly malignant tumor, ranking as the seventh most common cancer worldwide in terms of incidence (604,000 new cases in 2020) and sixth regarding overall mortality (544,000 deaths). This high mortality rate signifies one in 18 cancer-related deaths from EC^1^. The incidence of EC has dramatically increased in the past few decades and is expected to continue to rise, however, the etiology and pathology of EC remain poorly understood^2,3^. The prognosis of EC is dismal, with an estimated 5-year survival rate of only 15–20%, due to the extremely aggressive biology of EC, the difficulty of early diagnosis, and the limitations of effective treatment^4^. There are two major histological types of EC, including esophageal squamous cell carcinoma (ESCC) and esophageal adenocarcinoma, with distinct demographics, risk factors and clinical features^5,6^. Since inconspicuous symptoms and low sensitivity and specificity of biomarkers, most EC patients are already at an advanced stage when they are diagnosed. In clinical practice, multimodality therapy is widely used to treat EC, such as endoscopic resection, surgery, chemotherapy, radiotherapy, chemoradiotherapy, targeted therapy, or their combinations according to the stage of EC. Nevertheless, these treatments can only improve the survival and life quality of patients to a limited extent^7^. The increasing incidence and unimproved prognosis highlight the urgent need for etiologic and pathologic studies of EC, which will contribute to early diagnosis, patient classification, prognostic analysis, and especially for the discovery of promising targets to develop novel and more effective therapies.

More than 150 RNA modifications have been identified in organisms, including N6-methyladenosine (m6A), 5‑methylcytosine (m5C), N1-methyladenosine (m1A) and so on^8,9^. Among these modifications, m6A methylation is recognized as the most abundant endogenous modification of mRNA, accounting for 0.1–0.4% of total adenosine residues^10,11^. This reversible modification process is dynamically regulated by multiple m6A regulators, including methyltransferases (also known as “writers”), demethylases (“erasers”) and binding proteins (“readers”)^12,13^. In addition, increasing studies have demonstrated that m6A modification plays an essential role in various biological processes like cell differentiation, DNA damage response and circadian period^14,15^. Aberrant m6A modification has been identified to be associated with a variety of disorders such as dysregulation of cell differentiation and death, impaired self-renewal capacity, developmental defects and disturbed immune response^16,17^. Moreover, recent studies have reported that genetic mutation and aberrant expression of m6A regulators are implicated in human cancers, such as gastric cancer, colorectal cancer and breast cancer^18,19^. Thus, the in-depth research on m6A regulators and comprehensive analysis of m6A modification patterns may shed light on more effective and personalized therapies for EC patients.

In terms of the role of m6A modification in EC, an in vitro study suggested that methyltransferase-like 3 (METTL3) promotes cell proliferation and invasion partly by activating ATK signaling^20^. Additionally, Zhang et al. has indicated that platinum could increase METTL3-mediated m6A modification level by regulating SNHG3/miR‑186‑5p, which in turn enhances the efficacy of platinum^21^. And METTL3 expression is also associated with poor prognosis of ESCC patients^22^. There are several other m6A regulators that can affect EC tumorigenesis and progression, including ALKBH5^23^, FTO^24^, HNRNPA2B1^25^ and YTHDC2^26^. Interestingly, cigarette smoking could upregulate eEF2K expression and confer survival advantage to ESCC cells by reducing m6A modification of LINC00278^27^. These studies focusing on specific single m6A regulator have revealed novel potential mechanisms underlying EC pathogenesis. Nevertheless, comprehensive evaluation of multiple regulators is necessary to provide an overview of m6A modification pattern and uncover their integrated role in EC. In this study, we combined all available data from The Cancer Genome Atlas (TCGA) and Gene-Expression Omnibus (GEO) databases to describe the interactions between m6A regulators, identify different m6A modification patterns and correlate m6A modification pattern with immune cell infiltration within tumor microenvironment (TME). The m6A-related gene signatures were also analyzed. We further established a scoring system to quantitatively assess m6A modification pattern for individual patients, which may contribute to precise classification, personalized treatment and prognostic analysis of EC patients.

## Methods

### Dataset source and preprocessing of esophageal cancer

The public data of gene expression and full clinical annotation were downloaded from TCGA and GEO databases. Patients without survival information were excluded from further analysis. Gene expression data were downloaded from GEO database with two series (GSE53622 and GSE63524, https://www.ncbi.nlm.nih.gov/geo/) and TCGA database with one dataset (TCGA-ESCA, https://tcga-data.nci.nih.gov/tcga/). With regard to GEO datasets, the normalized matrix files with Transcripts Per kilobase Million (TPM) value were downloaded directly from GEO database. As for TCGA dataset, we used R package TCGAbiolinks, developed for integrative analysis of Genomic Data Commons (GDC) data, to obtain RNA sequencing data and fragments per kilobase of exon model per Million mapped fragments (FPKM) from GDC (https://portal.gdc.cancer.gov/)^28^. Based on the definitions of FPKM and TPM, the calculation of FPKM will first normalize for sequencing depth and then gene length, whereas TPM calculation dose the opposite order. This makes the sum of TPM values for all genes in each sample equal, thus TPM appears to be more suitable for comparing the differences in gene expression among different samples. In order to integrate the data from both databases for subsequent overall analysis, the FPKM value in TCGA database was transformed into TPM value. Additionally, this integrated matrix was then corrected for batch effects from non-biological technical bias using the ComBat function of sva package^29^. The primary parameters we adjusted for batch effects included specific variables (e.g., sequencing run date and experiment batch) and other covariates (e.g., age, gender and disease status). Besides, the somatic mutation data and Copy Number Variation (CNV) were downloaded from TCGA database. Survival data of EC patients and their phenotypic information were obtained from GEO and TCGA databases. Data analysis was performed with R (version 4.1.0) and R Bioconductor package^28^.

### Unsupervised clustering of m6A regulators

A total of 23 m6A regulators were collected from previous studies to characterize various m6A modification patterns, including 8 writers (METTL3, METTL14, METTL16, WTAP, VIRMA, ZC3H13, RBM15, RBM15B), 2 erasers (ALKBH5, FTO) and 13 readers (YTHDC1, YTHDC2, YTHDF1, YTHDF2, YTHDF3, HNRNPC, FMR1, LRPPRC, HNRNPA2B1, IGFBP1, IGFBP2, IGFBP3, RBMX). Based on the overall expression levels of these 23 m6A regulators, unsupervised clustering analysis was used to classify patients into different groups with distinct m6A modification patterns. Consensus clustering, a technique realized by the consensus package in R, provides a robust and consistent way to identify natural clusters within a dataset^30^. In short, a repeated sampling approach was used to draw 80% proportion of sample size from the original sample pool as subsets. Then, each subset was separately clustered using k-means as our base clustering method. The consensus matrix was generated by calculating the probability that any two samples would be classified into the same category during multiple clustering for subsets. In our study, 1, 000 repetitions of sampling subsets were performed to ensure the stability. The number of clusters was assigned from 1 to 9. To determine the optimal number of clusters, we examined the consensus or agreement across multiple clustering by observing the heatmap of consensus matrix. Moreover, the consensus cumulative distribution function (CDF) was plotted. The relative change in area under CDF curve was subsequently estimated when the number of clusters changed from 1 to 9. A clear "elbow" or inflection point in the plot of relative change in area under CDF curve suggests a specific clustering number. The principles to select an optimal number of clusters include stability of clusters, the elbow in the CDF plot and biological interpretability.

### Gene set variation analysis (GSVA) and functional annotation

We used GSVA R packages to perform GSVA enrichment analysis in a nonparametric and unsupervised mode^31^. The differences in biological processes and signaling pathways were investigated among clusters with distinct m6A modification patterns. In brief, genes were ranked according to their expression levels for each sample. In addition, genes were categorized into a specific gene set or a paired residual gene set. Then, the distribution scores were calculated for the specific gene set and the residual gene set, respectively. And the target gene set was assigned a score by comparing the deviation with the residual gene set. During GSVA process, we needed to input sample and gene expression matrix and list of gene sets to obtain the sample and gene set expression matrix. By transforming individual gene expression levels into the overall expression level of a gene set, we could better understand variations in gene set involved in certain cellular function, biological process and regulatory pathway. The typical gene sets were downloaded from Gene Ontology (GO) and Kyoto Encyclopedia of Genes and Genomes (KEGG)^32–34^ databases. An adjusted P value of less than 0.05 was considered statistically significant. Functional annotation for m6A-related genes was performed based on clusterProfiler R package^35^ and the FDR threshold was set at 0.05.

### Evaluation of immune cell infiltration within TME

The single-sample gene set enrichment analysis (ssGSEA)^36^ was utilized to evaluate the abundance of immune cell infiltration in TME of EC. ssGSEA is a computational algorithm that can determine an enrichment score of a predefined gene set for each individual sample. Since the absolute number of infiltrating immune cells was not available, we employed gene signatures to indirectly represent various immune cell types. In our study, the gene signatures were obtained from the research of a previous published article by Charoentong et al. (Supplementary Table S1)^37^. These gene signatures comprised genes known to be specifically expressed in certain immune cell types and could indicate the presence or activity of these cells in the sample. To explore the differences in the abundance for each type of TME cell infiltration among m6A clusters, we calculated the enrichment scores of gene signatures for each sample using ssGSEA analysis. Briefly, all genes were ranked according to their expression levels in each sample. The enrichment score for a designated gene set, which characterized a specific immune cell type, was then calculated based on the distribution of this gene set among the expression ranking table of all genes. Thus, this enrichment score could assess the relative abundance of the immune cell type defined by this gene set in TME infiltration.

### Identification of differentially expressed genes (DEGs) between m6A clusters

EC patients were classified into three m6A modification patterns by a comprehensive assessment of the expression of 23 m6A regulators. Empirical Bayesian approach of limma R package was used to identify pairwise DEGs by comparing these m6A clusters two-by-two. An adjusted P value < 0.001 was considered statistically significant in determining DEGs^38^. The pathways enriched in different clusters were further investigated by GO enrichment analysis and KEGG pathway analysis^32–34^.

### Generation of m6A-related gene signature

To quantitively assess m6A modification pattern for individual EC patients, we established a scoring system with reference to the study of Zhang et al.^39^. Briefly, EC patients were classified into distinct groups based on the overlapping DEGs extracted from the comparisons of any two m6A clusters. Subsequently, prognostic analysis was performed for each of these overlapping genes by a univariate Cox regression model. Genes with significant prognostic effects were selected for further principal component analysis (PCA). We calculated m6A score by the formula as below: m6A score = Σ (PC1_i_ + PC2_i_), where i represents the expression of m6A phenotype-associated genes and PC1 and PC2 mean principal component 1 and 2^40,41^.

### Statistical analysis

We performed one-way analysis of variance (ANOVA) followed by Holm-Sidak’s multiple comparison adjustment for data conforming to normal distribution and variance chi-square, otherwise we performed Kruskal–Wallis test. The mutation landscape was categorized into two groups with high and low m6A scores using maftools package. We also depicted the CNV landscape for 23 included m6A regulators via RCircos package^42^. Correlations coefficients between m6A regulator expression and TME immune infiltrating immune cells were calculated using Spearman and distance correlation analysis. With regard to the correlation between m6A score and survival of EC patients, survminer R package^43^ was applied to calculate the cut-off point. In order to classify EC patients based on m6A score, we tested all potential cut-off points to find the maximum log-rank statistic. Kaplan–Meier curves were produced for survival analysis and log-rank tests were conducted to determine the significance of differences. Both Univariate and multivariate Cox regression models were adopted to evaluate prognostic value of m6A regulators and m6A-related genes. Receiver operating characteristic (ROC) curve and area under the curve (AUC) were preformed to assess specificity and sensitivity. All statistical P values were bilateral and P value less than 0.05 was considered statistically significant if not otherwise stated. All data analysis was achieved in 4.1.0 version of R software.

## Results

### Gene variation landscape of m6A regulators

**Figure 1:**
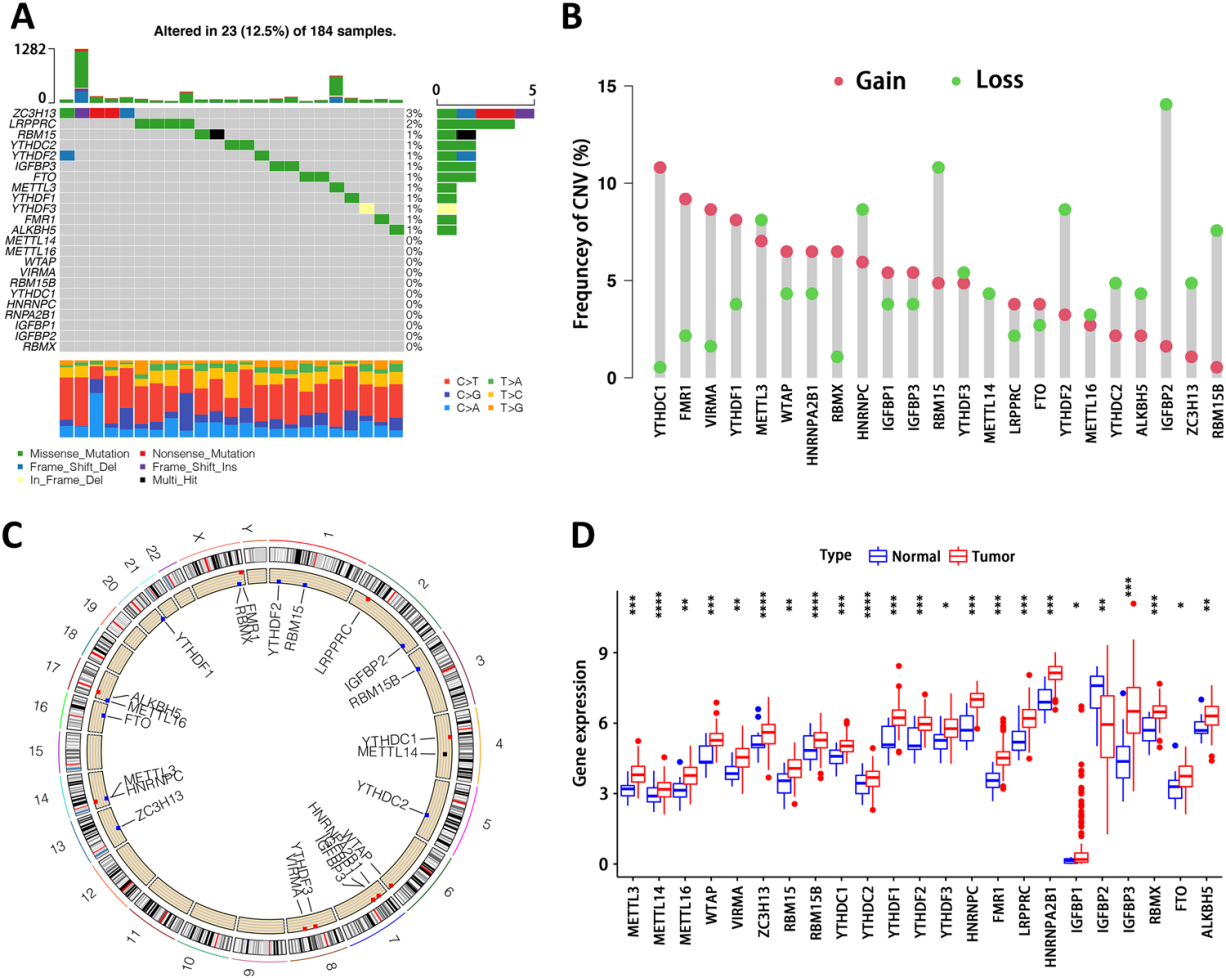
Characterization of 23 m6A regulators. (**A**) Mutations of included 23 m6A regulators. (**B**) Copy number variation (CNV) landscape of included 23 m6A regulators. (**C**) Location of m6A regulators with CNV alteration on chromosomes. (**D**) Gene expression of m6A regulators between tumor and normal samples. *p < 0.05, **p < 0.01, ***p < 0.001; ns, not significant.

A total of 23 m6A regulators were analyzed in this study (Supplementary Table S2), including 8 writers, 2 erasers and 13 readers. As shown in Fig. 1A, 23 out of 184 EC patients showed mutations of m6A regulators with a frequency of 12.5%. ZC3H13 exhibited the highest mutation frequency, followed by LRPPRC as the second. Two erasers, FTO and ALKBH5, both showed mutations. Meanwhile, nearly half of m6A regulators (11/23) were detected without mutations, including 5 writers (i.e., METTL3, METTL14, WTAP, VIRMA and RBM15B) and 6 readers (i.e., YTHDC1, HNRNPC, HNRNPA2B1, IGFBP1, IGFBP2 and RBMX). CNV alteration analysis indicated that all regulators experienced both gain and loss in copy number except METTL14, which had only CNV deletion (Fig. 1B). Besides, YTHDC1 and IGFBP2 showed the highest frequency of CNV amplification and deletion, respectively. We further investigated the location of m6A regulators with CNV alteration on chromosomes (Fig. 1C). Gene expression of almost all m6A regulators differed between tumor and normal samples (Fig. 1D). Thus, we could definitely distinguish tumor from normal tissues. We also explored the relationship between genetic variation (Fig. 1B) and expression (Fig. 1D) of m6A regulators. It was found that CNV alteration could affect the expression of m6A regulators. For instance, YTHDC1 with amplified CNV showed markedly higher gene expression in EC samples compared to normal samples, and vice versa (e.g., IGFBP2). It should be noted that gene expression of all m6A regulators, except IGFBP2, was increased in tumor samples, and most of them were statistically significant. Our results demonstrated a high degree of heterogeneity in genetic variation and gene expression of m6A regulators between tumor and normal tissues, suggesting an essential role of these regulators in EC occurrence and progression.

### m6A modification patterns mediated by 23 regulators

**Figure 2:**
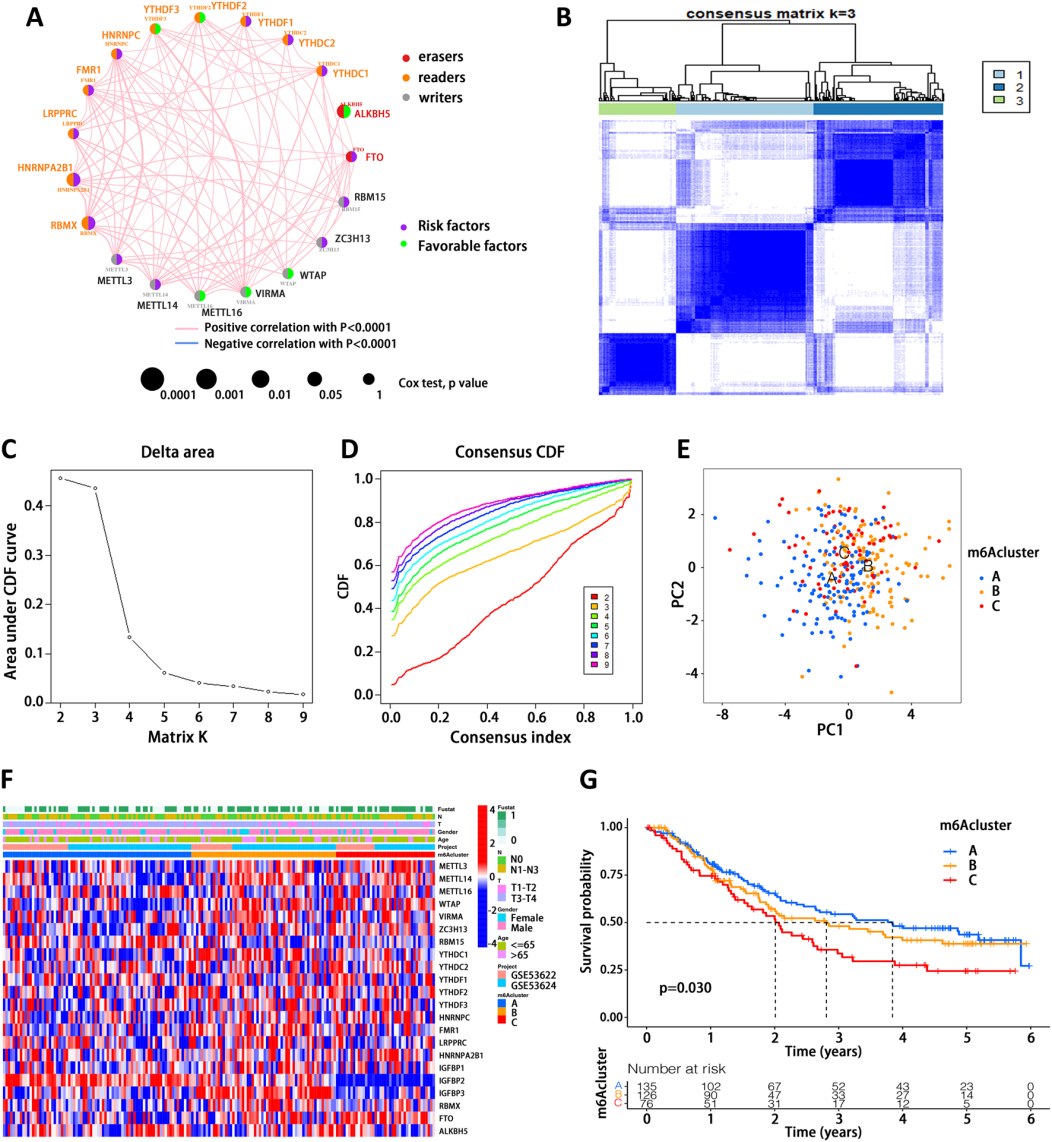
Identification of m6A modification patterns based on m6A regulators. (**A**) Interaction network among m6A regulators. (**B**) Consensus matrix when the number of clusters is 3. (**C**) Relative change in area under the cumulative distribution function (CDF) curve with different number of clusters. (**D**) Consensus CDF when the number of clusters is between 2 and 9. (**E**) Principal component analysis (PCA) of m6Aclusters. (**F**) Heatmap of gene expression of m6A regulators in distinct m6Acluster. (**G**) Prognostic analysis of these 3 m6Aclusters.

Clinical data from two GEO datasets and one TCGA dataset were included and combined into one cohort for subsequent analysis. The prognostic effect of each m6A regulator was investigated through a univariate Cox regression model (Supplementary Table S3) and those regulators with statistical significance were shown in Supplementary Fig. S1A–I. We also depicted an integrated landscape of interactions among m6A regulators (Supplementary Table S4). Interestingly, the interaction network indicated that all correlations among these regulators were positive (Fig. 2A). Besides, we examined whether gene mutations in one m6A regulator would affect gene expression of another regulator. We analyzed the differences in gene expression between wild and mutant type for three m6A regulators with the highest mutation frequencies, including ZC3H13, LRPPRC and RBM15. METTL14 expression was dramatically higher in ZC3H13 mutant type compared to that in wild type (Supplementary Fig. S1J). VIRMA was significantly upregulated in tumors with LRPPRC mutation (Supplementary Fig. S1K). A prominently elevated expression of IGFBP3 was observed in RBM15-mutant tumors compared to corresponding wild-type tumors (Supplementary Fig. S1L). Taken together, genetic mutations in all these 3 m6A regulators were related to higher expression of other specific regulators, suggesting a positive effect of mutation on gene expression. Based on the expression of these 23 m6A regulators, EC patients were classified into three groups with distinct m6A modification patterns using ConsensusClusterPlus R package. The consensus matrix was determined to be three, as the highest efficiency and least crossover among EC samples (Fig. 2B–D). Then, we performed principal component analysis to construct these 3 groups at the level of principal components 1 and 2, named m6Acluster A, m6Acluster B and m6Acluster C (Fig. 2E, Supplementary Table S5). In addition, heatmap was plotted to visualize the gene expression of m6A regulators in these 3 clusters (Fig. 2F). The overall expression of these 23 m6A regulators exhibited the highest level in m6Acluster B, followed by m6Acluster C, while lowest in m6Acluster A. Notably, IGFBP2 expression was obviously lower in m6Acluster C than the other two clusters. Prognostic analysis using Kaplan–Meier curves revealed significant differences in survival among these 3 clusters, with m6Acluster C being the worst (Fig. 2G).

### GO and KEGG analyses in three m6A clusters

To further explore the differences in biological features among these 3 m6A clusters, we conducted both GO and KEGG^32–34^ enrichment analyses by pairwise comparison. When comparing m6Acluster A with m6Acluster B, the top 20 terms of GO and KEGG analyses (Supplementary Table S6) were shown in Supplementary Fig. S2A,B, respectively. We found several prominent tumor-related signaling pathways like P53, WNT, TGF-β and T cell receptor (TCR) pathways enriched in KEGG analysis. Similarly, we performed the comparison between m6Acluster A and m6Acluster C by GO (Supplementary Fig. S2C) and KEGG (Supplementary Fig. S2D), and more detailed information was presented in Supplementary Table S7. Importantly, multiple immune-related pathways were enriched in both GO and KEGG analyses, including antigen processing and presentation, natural killer (NK) cell mediated immunity, T cell mediated immunity, Toll like receptor (TLR) signaling pathway and so on. Besides, we also compared m6Acluster B and m6Acluster C (Supplementary Table S8) and results from GO showed the term of esophageal atresia (Supplementary Fig. S2E) while KEGG analysis indicated enriched terms such as cell cycle, TGF-β, WNT and antigen processing and presentation (Supplementary Fig. S2F). Such distinct characteristics in these 3 m6A clusters would definitely increase our understanding of EC biology and contribute to the development of novel therapy.

### Landscape of immune cell infiltration in TME

**Figure 3:**
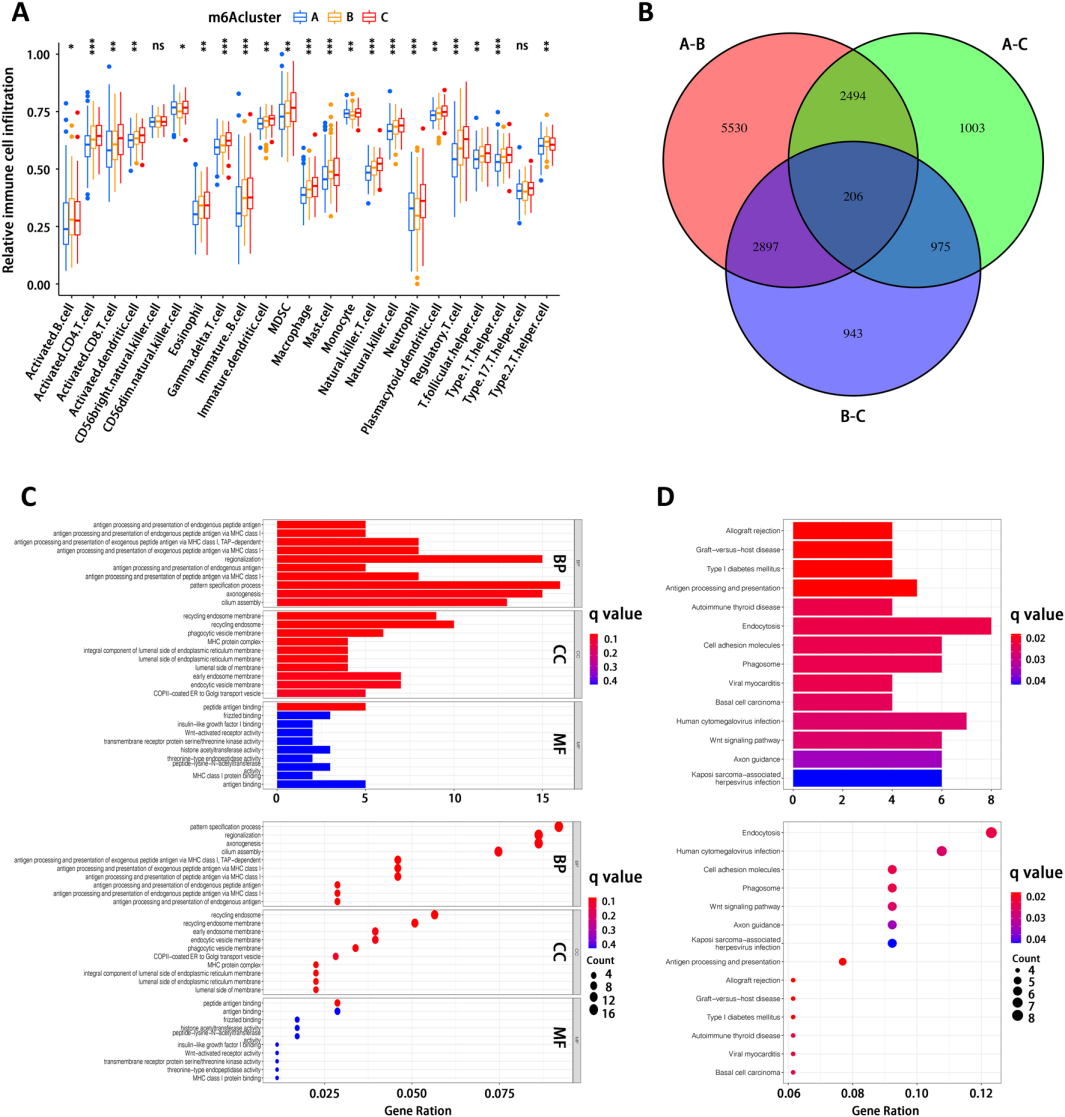
Landscape of immune cell infiltration in tumor microenvironment (TME) and identification of differentially expressed genes (DEGs) among m6Aclusters. (**A**) Infiltration of different types of immune cells in TME in 3 m6Aclusters. CDF, cumulative distribution function. (**B**) Identification of DEGs among m6Aclusters. (**C**) Gene Ontology (GO) enrichment analysis for the included 206 DEGs. *BP* biological process, *CC* cellular component, *MF* molecular function. (**D**) Kyoto Encyclopedia of Genes and Genomes (KEGG) enrichment analysis (www.kegg.jp/kegg/kegg1.html) for the included 206 DEGs.

Cellular components in TME, especially the infiltration of immune cells (Supplementary Table S1) was depicted and the majority of analyzed cell types exhibited remarkable differences among these 3 m6A clusters (Fig. 3A, Supplementary Table S9). For instance, immune cells were dramatically enriched in m6Acluster C, including CD4^+^ T cell, eosinophil, myeloid-derived suppressor cells (MDSC), macrophage and mast cell. However, the prognosis of patients in m6Acluster C did not show a matching advantage (Fig. 2G). Through literature search, we found previous studies that have reported an immune excluded phenotype, characterized by an abundant infiltration of immune cells. Nevertheless, these immune cells are mainly retained within the stroma surrounding tumor tissue rather than penetrating into tumor parenchyma. Moreover, stroma activation in TME is associated with the suppression of T cell^44^. In summary, our results indicated that m6A modification is closely associated with TME infiltration of immune cells, thus affecting tumor immune regulation and its response to immunotherapy.

### Characteristics and clustering of m6A-related genes

**Figure 4:**
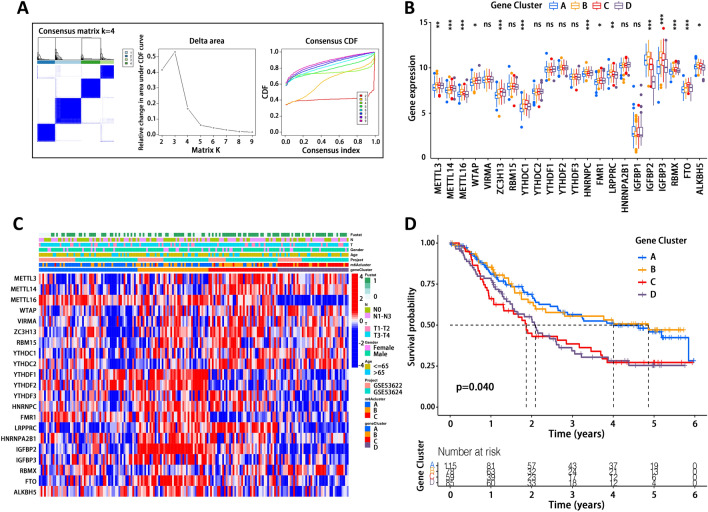
Characterization of gene clusters based on differentially expressed genes (DEGs). (**A**) Identification of gene clusters based on DEGs. (**B**) Gene expression of m6A regulators in each gene cluster. (**C**) Heatmap of gene expression of m6A regulators with clinical information of patients. (**D**) Prognostic analysis of these 4 gene clusters by Kaplan–Meier survival curve. *p < 0.05, **p < 0.01, ***p < 0.001; ns, not significant.

In order to further investigate m6A modification in EC, we identified 206 m6A-related DEGs among these 3 m6A clusters using limma package (Fig. 3B) and performed prognostic analysis for these 206 DEGs using a univariate Cox regression model (Supplementary Table S10). Then, we conducted GO and KEGG analyses for these 206 DEGs to reveal the enriched signaling pathways or biological processes. As for GO analysis, biological processes were mainly focused on antigen processing and presentation (Fig. 3C). Cellular component analysis showed that DEGs were primarily involved in recycling endosome, phagocytic vesicle and major histocompatibility complex (MHC) protein complex. Molecular functional analysis showed that DEGs were enriched in antigen binding, IGF, WNT and receptor protein kinase pathways. In addition, KEGG analysis^32–34^ also uncovered several signaling pathways including antigen processing and presentation, endocytosis, cell adhesion and phagosome (Fig. 3D). Interestingly, these DEGs and enriched pathways exhibited close association with immunity, which again suggested that m6A modification and m6A-related genes play a non-negligible role in immune regulation in EC. Moreover, we carried out unsupervised clustering analysis based on these 206 DEGs, dividing patients into 4 groups (Fig. 4A, Supplementary Table S11), which were named as gene cluster A-D. Gene expression of m6A regulators was analyzed in these 4 gene clusters (Fig. 4B), followed by clustering heatmap analysis (Fig. 4C). As shown in Fig. 4D, the patients in gene cluster A and B had a substantially better prognosis than those in gene clusters C and D.

### Establishment of m6A scoring system

**Figure 5:**
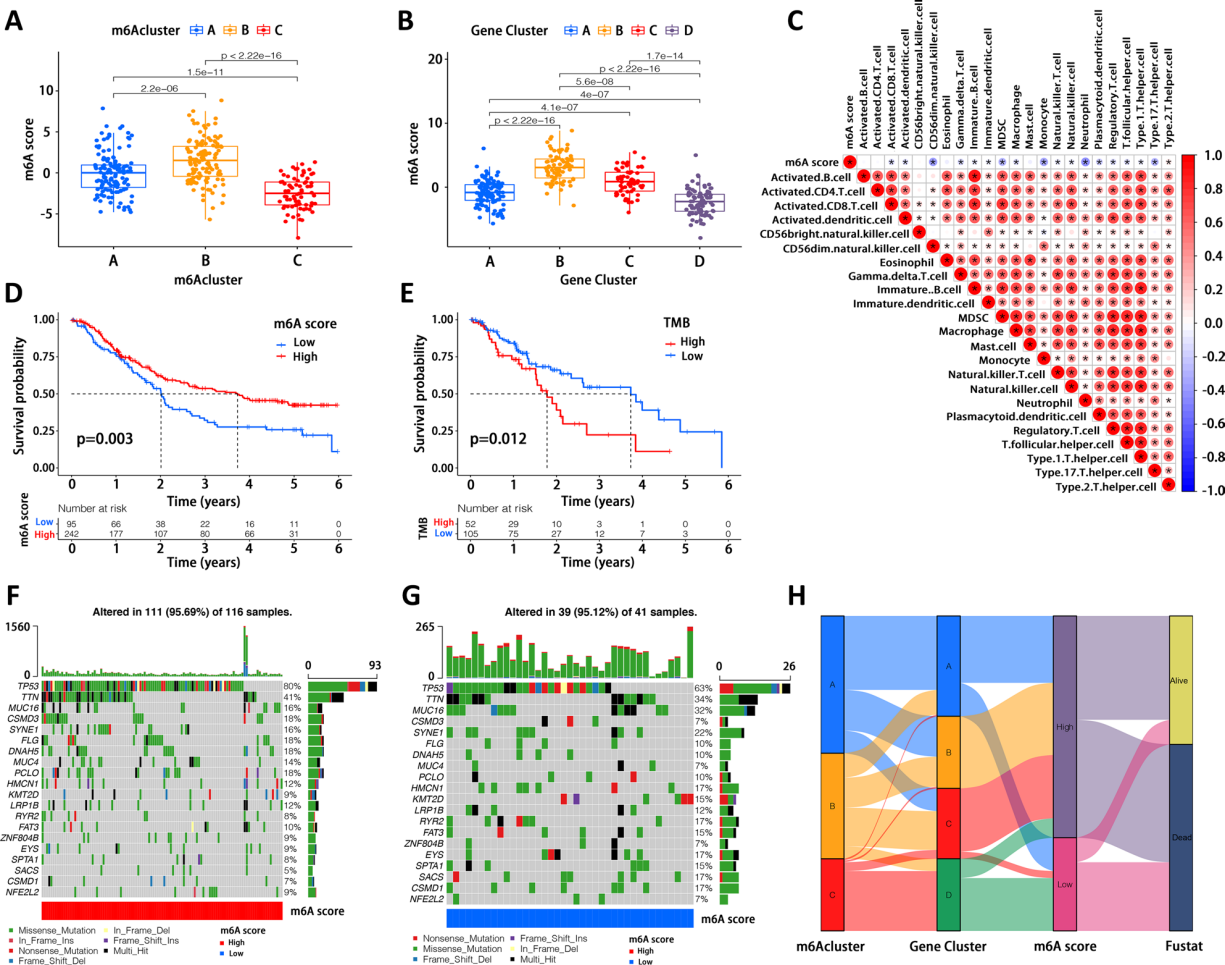
Establishment of m6A scoring system and tumor mutation burden (TMB) analysis. (**A**) Assessment of m6Ascore in different m6Aclusters. (**B**) Assessment of m6Ascore in different gene clusters. (**C**) Correlation between m6Ascore and immune cell infiltration. (**D**) Prognostic analysis for patients with high and low m6Ascore. (**E**) Prognostic analysis for patients with high and low TMB. (**F**,**G**) Analysis of gene mutations in patients with high (**F**) and low (**G**) m6Ascore, respectively. (**H**) Trajectory of each patient in m6Acluster, gene cluster, m6Ascore and survive classification by alluvial diagram.

The above results suggested a critical role of m6A modification in TME construction and immune landscape in EC. However, these analyses were based on patient populations rather than individual patients, which cannot fully capture individual diversity and heterogeneity and may be not appropriate and accurate to assess a particular patient. Thus, according to those m6A-related DEGs with significant impact on prognosis, we established a scoring system to quantitively evaluate m6A modification for individual patients, named as m6A score. It was found that m6Acluster B exhibited the highest m6A score while m6Acluster C was the lowest (Fig. 5A). Considering the worst survival in m6Acuster C, we hypothesized that a low m6A score predicts a poor prognosis. In addition, there was a significant difference in m6A score between any two gene clusters (Fig. 5B). Although the correlation between m6A score and survival in gene clusters was complex and multidimensional, we could observe that gene cluster D with the lowest m6A score had the poorest prognosis. We further illustrated the correlation between m6A score and TME cell infiltration (Fig. 5C) and indicated that almost all types of cell infiltration were negatively correlated with the m6A score, except type 2 T helper (Th2) cells. Consistently, EC patients in m6Acluster C, characterized by abundant immune cell infiltration and referred to as immune excluded phenotype, had the lowest m6A score as well as the worst prognosis. Importantly, EC patients with high m6A score showed a significant survival advantage compared to those with low m6A score (Fig. 5D). Additionally, we also examined whether tumor mutation burden (TMB) could affect the survival of EC patients. Previous studies have reported that TMB is a potential predictor of response to immunotherapy^45,46^. In our study, we demonstrated a remarkable survival benefit in the group with low TMB (Fig. 5E). Then, we further depicted the distribution of somatic mutations in groups with low and high m6A score, respectively (Fig. 5F,G). In both groups, the most prevalent mutation was *TP53*, which occurred in more than half of samples. The second highest mutation frequency was observed in *TTN*. The alluvial diagram visually depicted the attribution of each patient in categories of m6Acluster, gene cluster, m6A score and final outcome (Fig. 5H). Notably, almost all patients in m6Acluster C belonged to gene cluster D and presented low m6A score with dismal survival. Taken together, our results demonstrated that m6A score is a promising indicator for assessing immune infiltration and prognostic analysis.

### Prognostic analysis of patients with distinct clinical features

**Figure 6:**
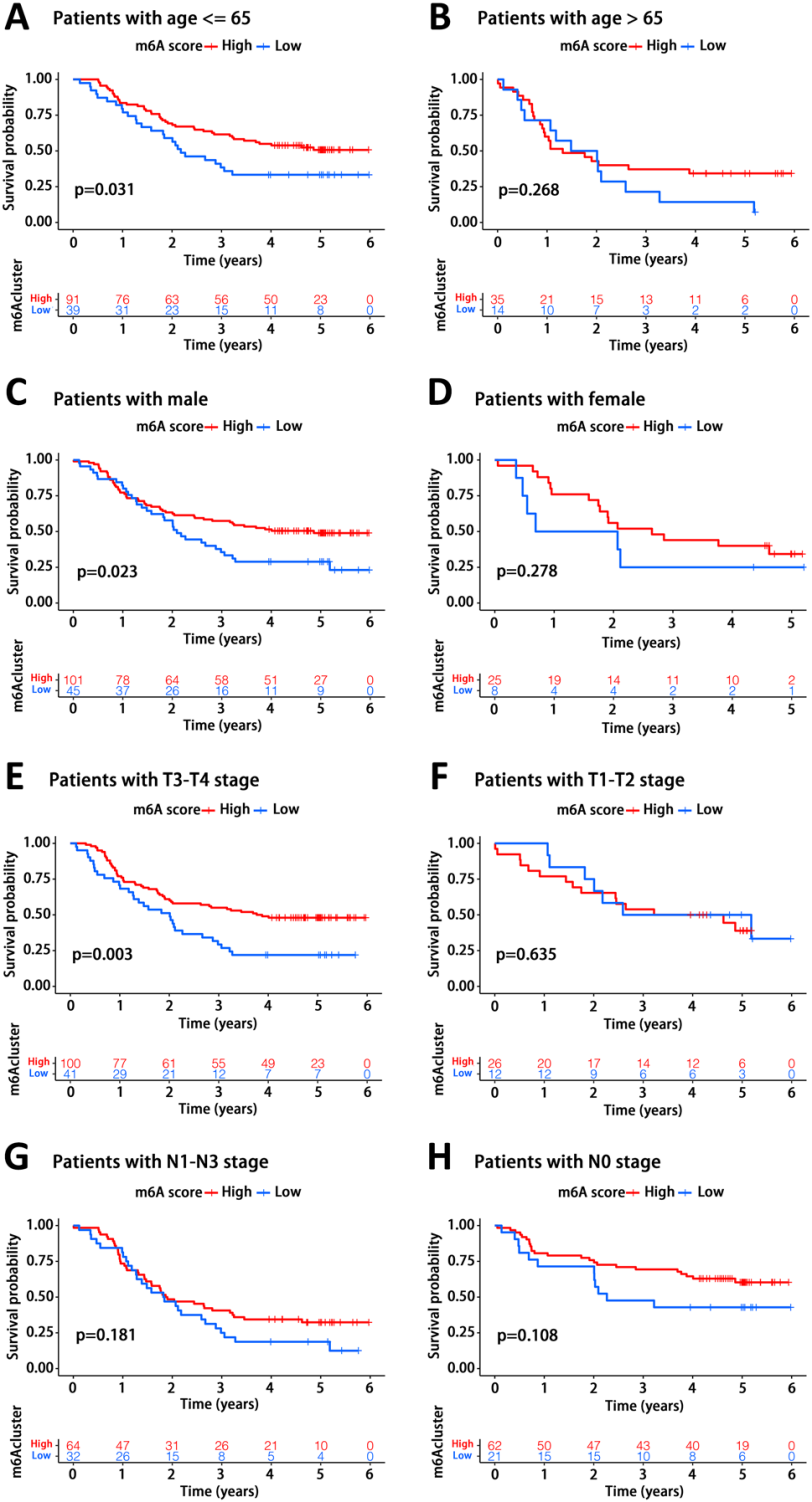
Prognostic analysis of subpopulation stratified by clinical features between patients with high and low m6A score. (**A**,**B**) Prognostic analysis of high and low m6Ascore groups in subpopulations older than 65 years (**A**) and younger (**B**). (**C**,**D**) Prognostic analysis of high and low m6Ascore groups in male (**C**) or female (**D**) patients. (**E**,**F**) Prognostic analysis of high and low m6Ascore groups in the subpopulations of T3-T4 stages (**E**) and T1-T2 stages (**F**). (**G**,**H**) Prognostic analysis of high and low m6Ascore groups in the subpopulations of patients with N1–N3 stages (**G**) and those with N0 stage (**H**).

To further verify the role of m6A score in the prognostic analysis of EC patients, we tested the survival difference between patients with high and low m6A scores in several specific subpopulation. Patients were classified into two subgroups according to different clinical features, including age, gender, tumor T stage and N stage, and then the distribution of m6A score between them was analyzed and presented in Supplementary Fig. S3A–D. It was found that there was no significant difference in m6A score in each pairwise comparison. Patients with high m6A score showed a remarkable survival advantage compared to those with low m6A score in subgroup younger than 65, while there was only a trend but no statistical difference in subpopulation older than 65 (Fig. 6A,B). In terms of gender, we found a dramatical survival benefit in male patients with high m6A score, however, no significant difference in female patients (Fig. 6C,D). As for tumor T stage, patients with high m6A score had a better prognosis than those with low m6A score in subgroup of T3-T4 stages, while a similar prognosis in subgroup of T1-T2 stages (Fig. 6E,F). Both subgroups of patients in N0 and N1-N3 stages, high m6A score tended to have a survival advantage despite no statistical significance (Fig. 6G,H). In general, patients with high m6A score in each subgroup appeared to have a better prognosis, whether statistically significant or not. Together, these results further demonstrated the critical role of m6A score in prognosis assessment.

## Discussion

With increasing incidence and poor prognosis, the management of EC has become a major clinical challenge. However, current clinical TNM staging system appears to be of limited value in guiding treatment and predicting prognosis of EC. Recent advances in the study of epigenetic modifications of RNA, including mRNA, have revealed that such modifications are closely involved in many biological processes and functions^8^. A growing number of mRNA modifications have been identified, some of which play a key role in cancers. Thus, research focusing on the mechanisms of mRNA modifications in tumorigenesis and progression may contribute to the discovery of novel targets for tumor therapy^47,48^. N6-methyladenosine (m6A), first discovered in the 1970s, is the most abundant internal modification on eukaryotic mRNA^9^ and plays an important role in cancer biology, as well as therapy resistance^49^. In the past decades, emerging evidence has indicated that m6A regulators were dysregulated in EC and revealed potential molecular mechanisms^20,27^. However, most studies focused only on a single m6A regulator and may not fully recognized the integrated role of m6A modification, mediated and coordinated by multiple m6A regulators.

In our study, we synthesized GEO and TCGA datasets to comprehensively investigate the role of 23 selected m6A regulators in EC, with the aim of peering into overall landscape of m6A modification pattern. We showed the mutation frequency of each m6A regulator, with ZC3H13 being the highest. Besides, we clarified the association between CNV alteration and gene expression level of these 23 m6A regulators. Generally, m6A regulators are upregulated in cancers, activating some oncogenes and thus promoting cancer progression^18^. Except IGFBP2, we observed an increased expression of other 22 m6A regulators in EC samples. This aberrant expression profiles could help us distinguish tumor from normal samples. In addition, IGFBP2 expression was significantly reduced in both m6Acluster C and gene cluster D, which had the lowest m6A scores and the worst prognosis. This result suggested that IGFBP2 may be an essential tumor suppressor gene in EC. As an mRNA binding protein, IGFBP2 plays a crucial role in cancer development and progression by regulating mRNA stability and localization^50^. Therefore, IGFBP2 may be a promising target and the underlying mechanism deserves further investigation in EC. Moreover, the network of interactions among m6A regulators showed that they were all positively correlated.

Based on the overall expression of these 23 m6A regulators, EC patients were classified into three groups with different m6A modification patterns using clustering analysis. There were significant differences in survival between m6A clusters, suggesting a predictive value of m6A modification for prognosis. The mainly enriched signaling pathways were identified, including immune-related pathways, such as antigen processing and presentation, T cell-mediated immunity and NK-mediated cytotoxicity. Then, we described the landscape of TME cell infiltration, especially immune cells, suggesting that m6A modification may be closely associated with tumor immune response, as well as therapeutic efficacy, including immunotherapy. Indeed, a growing number of m6A regulators have been demonstrated to be deeply implicated in immune escape and response to immunotherapy in various cancers through TME^51^. For instance, METTL3 accelerates M1 macrophages polarization by inducing the methylation of STAT1 mRNA^52^. With respect to acquired immunity, m6A modification can affect T cell homeostasis through regulating IL-7/STAT5/SOCS pathway^53^. Moreover, both single regulators such as ALKBH5^54^ and FTO^55^, and multiple regulators as a whole^56^ have been reported to affect the response to anti-programmed cell death 1 (PD-1) therapy. Besides, DEGs among m6A clusters were demonstrated and recognized as m6A-related genes. GO and KEGG analyses revealed that enriched signaling pathways were clearly involved in immunity, which confirmed again the great significance of m6A modification in immune regulation. Similar to the clustering of m6A modification phenotypes, patients were reclassified into different genomic subtypes based on the expression of DEGs. There was also significant difference in survival between these gene clusters.

Notably, immunotherapy has ushered in a new era of cancer treatment and rekindled hope for a cure of cancer^57^. However, it is unfortunate that current immunotherapies only show limited benefit and relatively low response in EC patients^58,59^, including targeted inhibition of programmed cell death 1 ligand 1 (PD-L1)/programmed cell death 1 (PD-1) and cytotoxic T-lymphocyte-associated antigen-4 (CTLA-4)^59^. In order to determine which patients will benefit most from certain immunotherapies, unremitting research is needed to further clarify the biology of EC, especially the role of immune regulation and surrounding microenvironment. TME consists of various cell populations (such as tumor cells, infiltrating immune cells and stromal cells), secreted cytokines and structural molecules that together participate in tumor development and progression, and also affect the response to immunotherapy. Given that TME is associated with EC resistance to conventional chemoradiation, preclinical trials have been conducted to overcome therapeutic resistance by targeting TME^60^. Moreover, Zhang et al. reported that m6A modification is closely related to TME immune infiltration features, according to which gastric cancer could be classified into different immunophenotypes^39^. Thus, it is promising to develop novel immunotherapies through comprehensive analysis of m6A regulator-mediated methylation modification patterns^55^.

Considering individual heterogeneity among EC patients, the above population-based analyses were limited to evaluating a specific patient. Therefore, we established a scoring system to quantitatively assess the m6A modification pattern of individual patients. Both in m6Aclusters and gene clusters, patients with the lowest m6A score experienced the worst survival. Additionally, m6A score was determined as an independent prognostic factor in EC patients. Our results showed that m6A score was negatively related to the infiltration of all TME immune cell types except Th2 cells. Patients in m6Acluster C had the lowest m6A score, the most abundant immune cell infiltration and the worst prognosis, suggesting an immune excluded phenotype. Patients in m6Acluster C had the lowest m6A score, the most abundant immune cell infiltration and the worst prognosis, suggesting an immune excluded phenotype. This phenotype has been proposed by the study from Chen et al.^44^ and also identified in the subsequent researches on gastric cancer^39^ and colon cancer^61^. Despite the abundance of immune cells, these immune cells cannot penetrate the tumor parenchyma but instead are maintained within the surrounding stroma. The stroma may be confined to the tumor capsule or may even penetrate the tumor parenchyma, making it appear as if the immune cells are actually inside the tumor^62,63^. Moreover, stroma-related T cells display proliferation and activation but not infiltration after anti-PD-L1/PD-1 therapy^64^. These findings imply that immune cells in the stroma may have generated an anti-tumor response but were ineffective because they failed to penetrate into the tumor parenchyma but remained in the stroma. Therefore, the key to treatment against this type of tumor is to overcome the rate-limiting step of immune cell penetration, especially T cell migration out of stroma. This m6A scoring system is promising to assess m6A modification pattern, characterize immune infiltration and guide personalized treatment and prognostic prediction. Therefore, more research is needed to further elaborate the correlation between m6A score and immune infiltration feature and to provide new insights into EC biology and even potential targets for developing novel therapies.

There were several limitations in our study. The data we analyzed were derived from public databases, focusing on the level of gene expression. However, actual protein expression, posttranslational modifications and subcellular localization of proteins will affect their biological behavior and function. More clinical samples and basic experiments are thus needed to further confirm the results in this study. Besides, the number of EC patients included in the analysis of this study is not large. Due to the lack of an absolute number of immune cells within TME, we could only indirectly assess the relative infiltration of these immune cells by evaluating the overall expression level of the gene sets that characterize specific immune cell types. In addition, we used the surv_cutpoint function from survminer R package to find the “best cutoff” based on the maximum log-rank statistics. It might be worthwhile to examine more threshold values, considering more factors relevant to clinical practice beyond algorithm, in order to identify an optimal cutoff for a more applicable and rational stratification strategy.

## Conclusions

In summary, our study investigated the expression profile of 23 m6A regulators in EC and demonstrated that their dysregulation was associated with patient survival. Three m6A modification patterns were identified with distinct characterization of TME cell infiltration as well as different prognosis. We established an m6A scoring system to quantitatively assess m6A modification for individual patients, which might contribute to precise classification, personalized treatment and prognostic prediction for EC patients. In addition, m6A regulators or m6A-related genes are potential targets and provide additional candidates for the development of novel therapeutic strategies, with the purpose of improving the survival of EC patients.

### Supplementary Information

Supplementary Figure 1.

Supplementary Figure 2.

Supplementary Figure 3.

Supplementary Tables.

## Data Availability

Publicly available datasets were analyzed in this study and can be found here: GSE53622 and GSE63524, https://www.ncbi.nlm.nih.gov/geo/; TCGA-ESCA, https://tcga-data.nci.nih.gov/tcga/.

## Abbreviations

EC: Esophageal cancer
ESCC: Esophageal squamous cell carcinoma
EAC: Esophageal adenocarcinoma
m6A: N6-methyladenosine
m5C: 5‑Methylcytosine
m1A: N1-methyladenosine
PD-L1: Programmed cell death 1 ligand 1
PD-1: Programmed cell death 1
CTLA-4: Cytotoxic T-lymphocyte-associated antigen-4
TME: Tumor microenvironment
TCGA: The cancer genome atlas
GEO: Gene-expression omnibus
FPKM: Fragments per kilobase of exon model per million mapped fragments
CNV: Copy number variation
GSVA: Gene set variation analysis
GSEA: Gene set enrichment analysis
DEGs: Differentially expressed genes
GO: Gene ontology
KEGG: Kyoto encyclopedia of genes and genomes
PCA: Principal component analysis
MDSC: Myeloid-derived suppressor cells
TMB: Tumor mutation burden
