# Supplementary material for: m6A regulator-mediated methylation modification patterns and tumor microenvironment immune infiltration with prognostic analysis in esophageal cancer
Source: Sci Rep. 2023 Nov 11;13:19670. doi: 10.1038/s41598-023-46729-1 (PMC10640615; doi:10.1038/s41598-023-46729-1)
Supplement: Supplementary file 1 — Supplementary Figure 1. [file 41598_2023_46729_MOESM1_ESM.pdf]

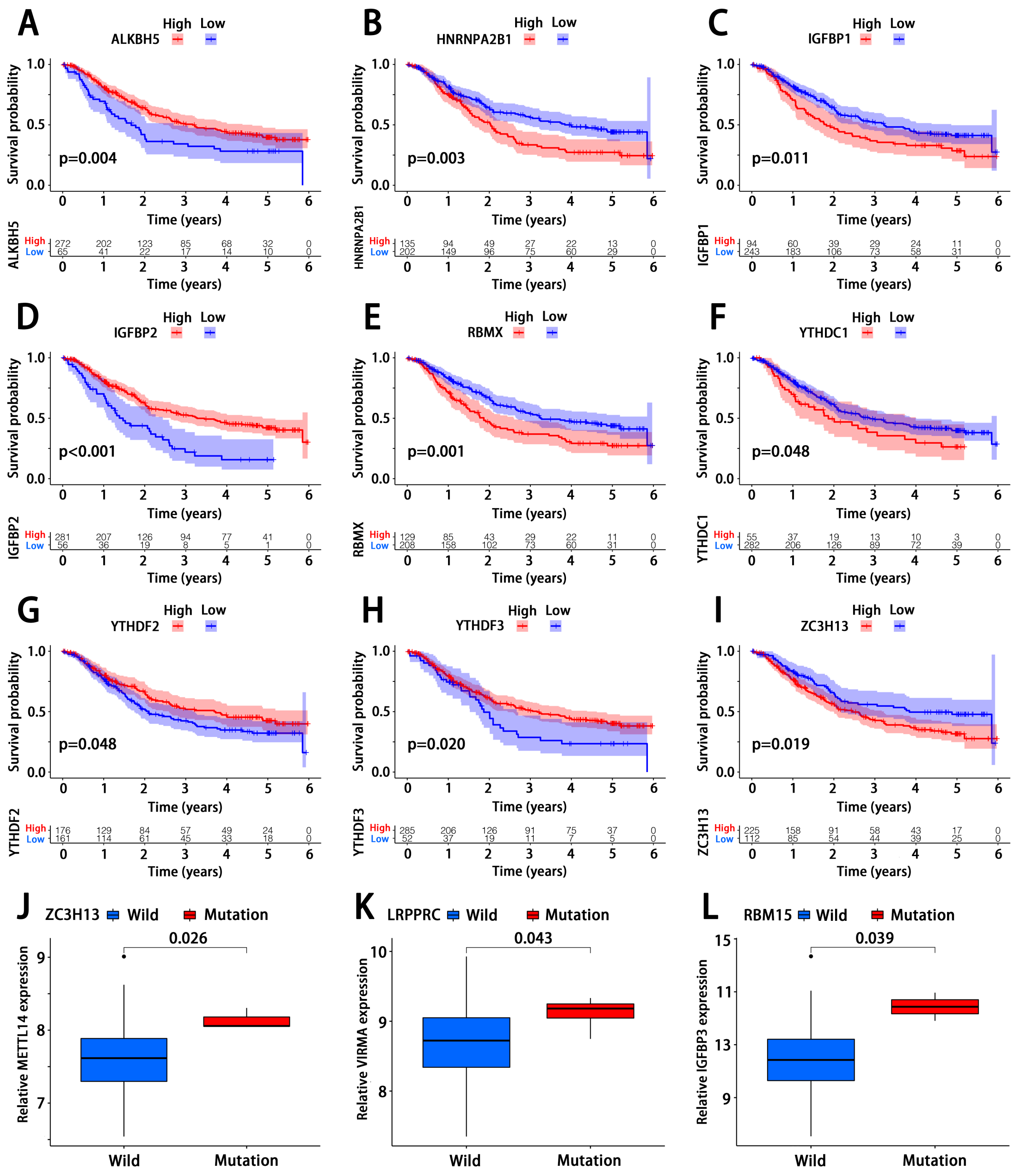

Supplementary Figure S1. Prognostic analysis for m6A regulators and correlation among m6A regulators. (A-I) The effect of m6A regulators on survival with statistical significance, including ALKBH5 (**A**), HNRNPA2B1(**B**), IGFBP1(**C**), IGFBP2 (**D**), RBMX (**E**), YTHDC1 (**F**), YTHDF2 (**G**), YTHDF2 (**H**) and ZC3H13 (**I**). **(J)** METTL14 expression in ZC3H13 wild-type and mutant groups. **(K)** VIRMA expression in groups of LRPPRC wild and mutant types. **(L)** IGFBP3 expression in RBM15 wild-type and mutant patients.
