# Supplementary material for: m6A regulator-mediated methylation modification patterns and tumor microenvironment immune infiltration with prognostic analysis in esophageal cancer
Source: Sci Rep. 2023 Nov 11;13:19670. doi: 10.1038/s41598-023-46729-1 (PMC10640615; doi:10.1038/s41598-023-46729-1)
Supplement: Supplementary file 2 — Supplementary Figure 2. [file 41598_2023_46729_MOESM2_ESM.pdf]

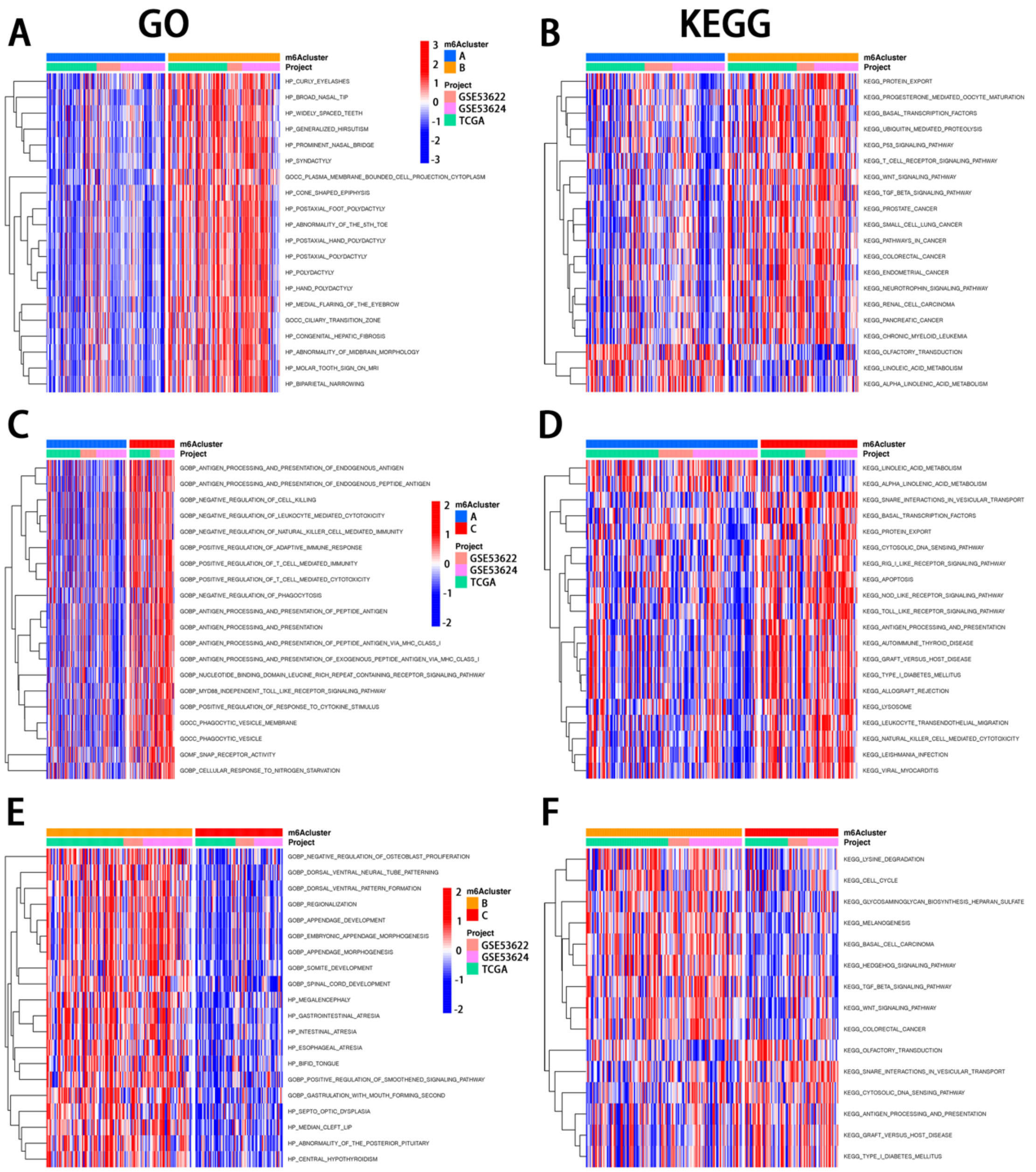

Supplementary Figure S2. Gene Ontology (GO) and Kyoto Encyclopedia of Genes and Genomes (KEGG) analyses among three m6A clusters. (A) GO analysis between m6Acluster A and m6Acluster B. (B) KEGG analysis between m6Acluster A and m6Acluster B. (C) Enrichment analysis of GO dataset by comparing m6Acluster A with m6Acluster C. (D) Enrichment analysis of KEGG dataset by comparing m6Acluster A with m6Acluster C. (E) GO enrichment analysis for the comparison between m6Acluster B and m6Acluster C. (F) KEGG enrichment analysis (www.kegg.jp/kegg/kegg1.html) for the comparison between m6Acluster B and m6Acluster C.
