# Supplementary material for: m6A regulator-mediated methylation modification patterns and tumor microenvironment immune infiltration with prognostic analysis in esophageal cancer
Source: Sci Rep. 2023 Nov 11;13:19670. doi: 10.1038/s41598-023-46729-1 (PMC10640615; doi:10.1038/s41598-023-46729-1)
Supplement: Supplementary file 3 — Supplementary Figure 3. [file 41598_2023_46729_MOESM3_ESM.pdf]

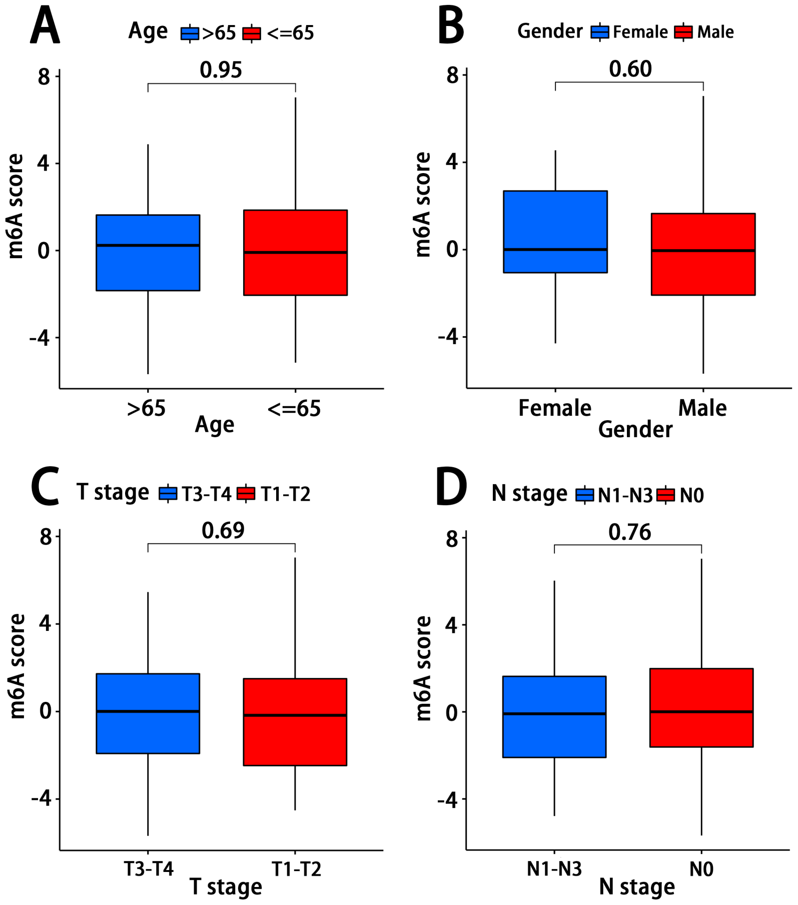

Supplementary Figure S3. Evaluation of m6Ascore in subpopulations with distinct clinical features. (A) Analysis of m6Ascore in patients older than 65 years or not. **(B)** The comparison of m6Ascore between male and female patients. **(C-D)** The effect of clinical T (**C**) and N (**D**) stage on m6Ascore.
